# Erosion behaviour of human, bovine and equine dental hard tissues

**DOI:** 10.1038/s41598-023-46759-9

**Published:** 2023-11-10

**Authors:** S. Hertel, S. Basche, V. Schmidt, C. Staszyk, C. Hannig, T. Sterzenbach, M. Hannig

**Affiliations:** 1 Clinic of Operative Dentistry, Medical Faculty Carl Gustav Carus, Technische Universität Dresden, Fetscherstraße 74, 01307 Dresden, Germany; 2 Clinic of Operative Dentistry, Periodontology and Preventive Dentistry, University Hospital, Saarland University, 66421 Homburg, Germany; 3 Institute for Veterinary-Anatomy, -Histology and -Embryology, Faculty for Veterinary Medicine, Justus-Liebig-University Giessen, Frankfurter Str. 98, 35392 Giessen, Germany

**Keywords:** Clinical trial design, Dental diseases

## Abstract

Dental hard tissues from different species are used in dental research, but little is known about their comparability. The aim of this study was to compare the erosive behaviour of dental hard tissues (enamel, dentin) obtained from human, bovine and equine teeth. In addition, the protective effect of the pellicle on each hard tissue under erosive conditions was determined. In situ pellicle formation was performed for 30 min on enamel and dentin samples from all species in four subjects. Calcium and phosphate release was assessed during 120 s of HCl incubation on both native and pellicle-covered enamel and dentin samples. SEM and TEM were used to examine surface changes in native enamel and dentin samples after acid incubation and the ultrastructure of the pellicle before and after erosive exposure. In general, bovine enamel and dentin showed the highest degree of erosion after acid exposure compared to human and equine samples. Erosion of human primary enamel tended to be higher than that of permanent teeth, whereas dentin showed the opposite behaviour. SEM showed that eroded equine dentin appeared more irregular than human or bovine dentin. TEM studies showed that primary enamel appeared to be most susceptible to erosion.

## Introduction

The characterisation of the pellicle as a mediator in the interactions between the tooth surface and the surrounding fluids is fundamental to basic dental research for the prevention of caries and erosion. The protective properties of pellicles against bacterial or erosive demineralisation of dental hard tissues have been demonstrated in numerous studies, as well as their targeted modification in mouth rinses to enhance their protection^1–4^. These studies have generally been carried out in a well-established in-situ-model designed to mimic real-life conditions. To this end, tooth samples (enamel or dentin) are exposed on splints in the oral cavity of volunteers. The use of non-human dental hard tissues for clinical investigations of intraoral bioadhesion processes is common in this kind of study. While some studies use dental hard tissues of human origin^5^, most investigations tend to use bovine material^4,6,7^.

There are many advantages to using bovine enamel and dentin samples. Obtaining human tooth tissue is very time consuming and costly, whereas bovine teeth are readily available from abattoirs. In addition, the large size of bovine incisors compared to human incisors makes is easy to obtain samples from the labial surfaces of anterior teeth using trepan burs. The crystallite arrangement and calcium content of bovine enamel is quite similar to that of human enamel^8^. The gradual decrease in calcium content from the enamel surface to the enamel-dentin interface in human enamel is also similar in bovine enamel^9^. However, bovine and human enamel differ in a number of ways. The crystallites of bovine enamel are wider and there is a lager interprismatic space^8,10^. In addition, bovine enamel has a lower fluoride concentration and higher porosity^5^. There is some debate in the literature as to whether the similarities between bovine and human dental hard tissues outweigh the differences to justify their use as a surrogate for human dental hard tissues, particularly when studying erosion behaviour. Turssi et al*.* used randomised cross-over studies to investigate whether the microhardness of enamel and root dentin in bovine teeth was similar to that of human teeth. They found that the microhardness values of bovine enamel were indistinguishable from those of the human counterpart, while root dentin did not appear to be a viable alternative to the human counterpart as its microhardness was significantly lower^8^. This finding is consistent with a study by Schielke et al*.* who used scanning electron microscopy to demonstrate that the number and diameter of dentinal tubules per square millimetre in bovine coronal dentin was not significantly different from that in human primary and permanent molars, whereas the tubule density in bovine root dentin was significantly higher. It was concluded that bovine coronal dentin, but not root dentin, was a suitable substitute for human molar dentin^11^. Wegehaupt et al*.* compared the dentin wear of primary and permanent human and bovine teeth using a combined erosion and abrasion test. No statistically significant difference in dentin wear was found between human third molars and bovine mandibular permanent incisors, justifying the use of bovine mandibular incisors as a substitute for human permanent teeth for further investigation in erosion and abrasion studies. However, differences in dentin wear have been reported between primary human and bovine teeth^12^.

A problem with bovine teeth arose in in-situ trials from concerns about possible health risks associated with bovine spongiform encephalopathy (BSE). In addition, bulls are typically slaughtered at a young age of less than 20 months and have no or up to two permanent incisors per quadrant (I1 and I2). Therefore, equine teeth were considered in the search for possible alternative animal tooth structure substitutes for future studies. Equine incisors are hypsodont, i.e. they have a long crown. They are characterised by an enamel layer covered by a thin layer of cement and have an enamel indentation (infundibulum) on their broad occlusal surface^13^. Throughout their lives, horses' teeth are subject to constant wear and tear. The loss of tooth structure on the occlusal surface is compensated for by prolonged elongation of the crown from the apical end of the tooth. This is facilitated by post-eruptive enamel deposition and secondary dentin apposition until the tooth reaches a post-eruptive age of approximately 5 years^13^. After this time, root formation begins. In addition to prolonged elongation of the tooth, the tooth erupts at a high rate throughout life to compensate for occlusal wear of 2–4 mm per year. However, although equine teeth are classified as hypsodont (long-crowned), occlusal wear outweighs tooth elongation over time and the tooth wears out. A critical feature of the equine pulp is the sufficient production of a special type of secondary dentin on the occlusal aspect to avoid pulpal exposure due to physiological tooth wear^14^. Typical of equine teeth is the formation of enamel folds adjacent to cementum and dentin formations. This increases the chewing surface and reduces the rate of wear because hard and soft dental materials are juxtaposed. The wear of incisors is less than that of molars due to their curved shape and lower load^13,14^. Ultrastructural studies have defined three types of equine enamel, classified according to the transverse appearance of the enamel prisms and the amount and appearance of interprismatic enamel^15^. To date, no study has been carried out on the suitability of equine teeth as an alternative to human tooth specimens for the study of surface erosion processes.

However, direct comparisons for the evaluation of erosion models, comparing not only species but also permanent and primary dentition, are rare and the experimental criteria are often very different^5^. To answer the question of whether bovine or equine teeth are a valid substitute for human teeth in both preclinical and clinical trials, it is important to consider not only the morphological and histological characteristics of the individual tissues, but also the effect of the pellicle formed in situ. It is important to investigate whether the protective effect of the pellicle on tooth hard tissues is the same in different species.

The aim of the study was to systematically investigate the demineralisation behaviour of the enamel and dentin of primary, permanent, bovine and equine teeth and to determine for the first time the protective effect of the in situ formed pellicles on the respective hard tissues under erosive conditions. In addition, electron microscopic micrographs will be used to characterise the morphological properties of the tooth hard substances after exposure to acid. This will provide reference data for future studies on demineralisation and erosion processes using bovine or equine dental hard materials.

## Material und methods

### Specimens preparation

**Figure 1:**
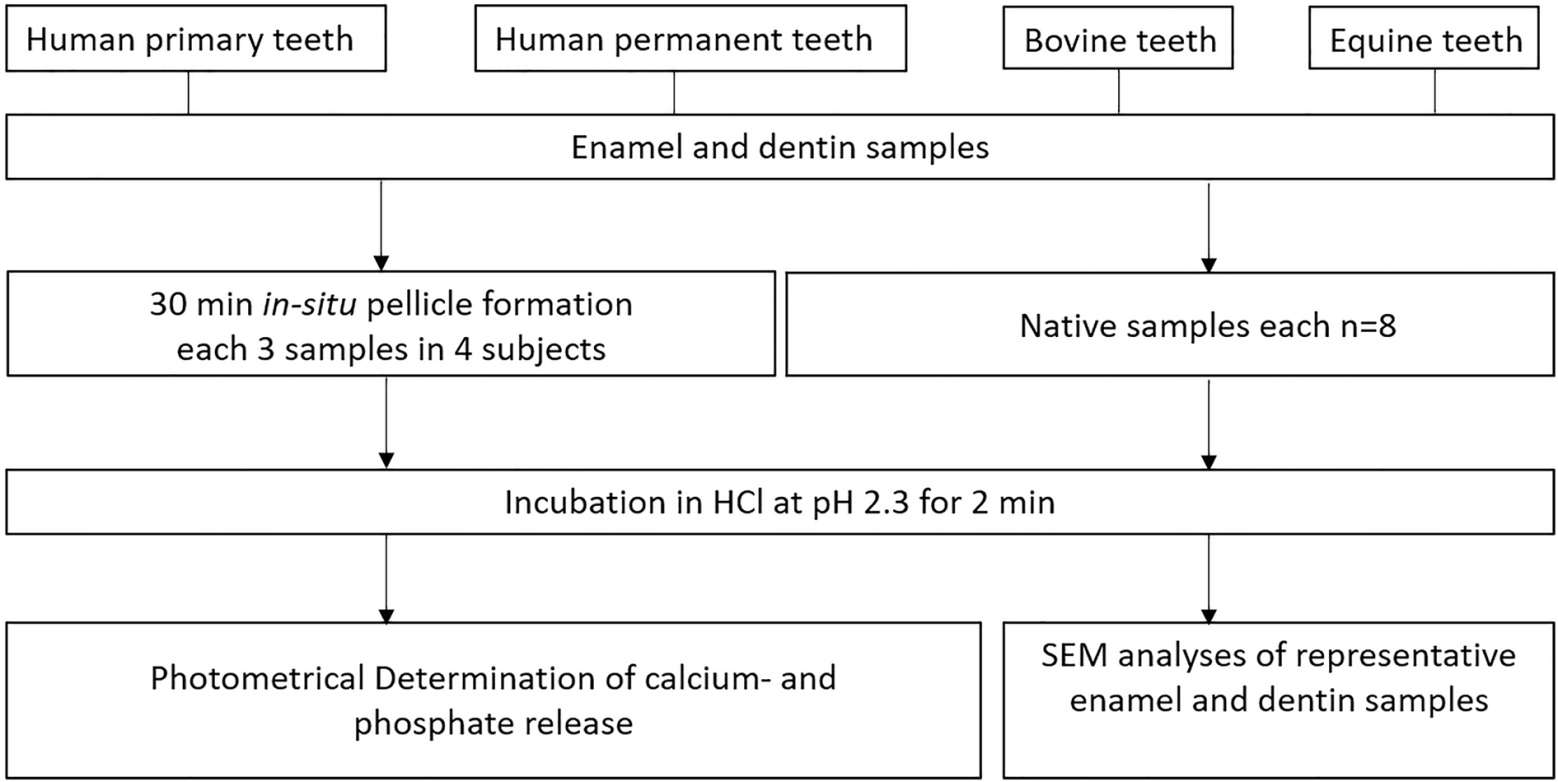
Flowchart of the experimental setup.

The present study included both in situ and subsequent ex vivo-investigations (Fig. 1). The ethics committee of the TU Dresden (EK 475112016) approved the study design. The adoption of bovine teeth was checked and approved by the Landesdirektion Sachsen, veterinary office. The present study included both in situ and subsequent ex vivo studies (Fig. 1). The study design was approved by the Ethics Committee of the TU Dresden (EK 475112016) and all methods were carried out in accordance with the relevant guidelines. The use of bovine teeth was reviewed and approved by the Regional Veterinary Office of Saxony. The non-resorbed bovine permanent incisors were obtained from BSE-negative 2–3 year old cattle from a slaughterhouse in Freiburg/Breisgau, Germany.

The equine incisors were provided by the Institute of Veterinary Anatomy, Histology and Embryology, Faculty of Veterinary Medicine, Justus Liebig University, Giessen, Germany. They came from horses aged between 15 and 30 years.

The human tooth samples came from primary and permanent molars extracted at the Dental Clinic of the University Hospital of Dresden, Germany.

First, enamel and dentin samples were taken from the labial surfaces of the animals' incisors using a trepan bur (Brasseler Komet, Lemgo, Germany). Both human primary and permanent enamel and dentin samples and equine hard tooth samples had a defined diameter. The thickness of the enamel samples was 2 mm. Dentin samples were obtained by grinding the enamel surface to expose the dentin and the dentine-enamel junction. Analogous to the established in-situ model from previous studies, the specimens were etched for 30 s at all sites except the outer enamel or dentin surface with 37% phosphoric acid gel (Scotchbond Universal Etchant, 3M ESPE, Neuss, Germany). They were then sealed with Optibond Primer (Kerr, Karlsruhe, Germany) for 30 s before applying Optibond Adhesive and light curing for 30 s in a halogen light furnace^3,4^. In the next step, the smear layer of the enamel samples was removed by incubation in sodium hypochlorite (3%) for 3 min. The samples were washed twice in ice-cold distilled water for 5 min using ultrasound and finally disinfected in ethanol (70%) for a further 10 min. Dentin samples were disinfected in ethanol (70%) for 1 min. They were then washed in distilled water for 2 × 5 min and then incubated in 3% EDTA for 1 min. Both enamel and dentin samples were then washed again for 2 × 5 min in distilled water and stored for 24 h at 4 °C in freshly distilled water before exposure in the oral cavity.

### Intraoral exposure

Four healthy volunteers aged between 29 and 48 years gave written consent to participate in the study. A dental examination confirmed that none of the subjects had any signs of caries, periodontal disease or unphysiological salivary flow.

For in situ pellicle formation on the test specimens, individual maxillary splints were made for each participant. Three enamel and three dentin specimens of each species were then mounted on the splints using polyvinylsiloxane impression material (President Light) so that they were exposed buccally in regions 14–16 and 24–26. The in situ pellicles were set for 30 min. During this time, the participants had to refrain from eating and drinking. Native enamel and dentin plates of each species, not exposed in the oral cavity, served as controls^3,4^.

### Ex-vivo erosion and photometric determination of calcium and phosphate release

For the ex vivo erosion test, the specimens were embedded in silicone impression material at the bottom of a test tube. A volume of 1 ml hydrochloric acid with a pH of 2.3 was then added to each of two specimens. During the 120-s incubation period, regular lifting strokes were performed with a 100 µl pipette to ensure constant acid application. Every 15 s, a volume of 100 µl of the acid was removed for photometric analysis and replaced with 100 µl of fresh acid. Mineral loss was determined by measuring calcium and phosphate release into solution. Therefore, photometrical assays based on the arsenazo III method (Calcium As FS, Holzheim, Germany) and the malachite green assay was applied^16,17^. By binding to calcium, arsenazo III forms a bluish-violet complex in an acidic environment. Malachite green forms a colour complex with phosphate. Specifically, a volume of 10 µl of each sample was added with 100 µl of arsenazo III reagent for calcium determination and 200 µl of malachite reagent for phosphate determination^18^. After 5 min (Ca) or 15 min (P), the absorbance at λ = 650 nm was recorded and concentrations of Ca or P were determined according to standard curves. This procedure was always carried out in triplicate. The calcium and phosphate release was based on the mean photometric absorbance values for the samples and their surface areas.

### Electron microscopic examination

#### Scanning electronic microscopy

Native enamel and dentin specimens of each species were fixed in 2.5% glutaraldehyde solution for 1 h. The specimens were then washed 5 times with phosphate buffered saline for 10 min each. The samples were then dehydrated in an ascending series of ethanol and carbon sputter coated. The samples were analysed in an ESEM XL 30 FEI scanning electron microscope (FEI, Eindhoven, The Netherlands) at a magnification of 25 to 20,000.

#### Transmission electron microscopy

To investigate the ultrastructure of pellicles on the enamel surfaces of human, bovine and equine teeth, a 2-h in situ pellicle was obtained from two subjects on the respective enamel surfaces and examined by transmission electron microscopy.

In order to visualise the erosive effects on the pellicle and enamel surfaces, a follow-up experiment was carried out. First, an in-situ pellicle was formed on the enamel samples of both subjects for 30 min. The pellicle-covered samples were then washed in vitro for 1 min in Coca-Cola Zero (The Coca-Cola Company, Erfrischungsgetränke AG, Berlin) at pH 2.2 and then exposed to the oral cavity for a further 89 min.

The pellicle-covered enamel specimens were removed from the splints and fixed in 2.5% glutaraldehyde/1.5% formaldehyde for 1 h. This was followed by post-fixation in osmium tetroxide for 2 h. After dehydration through an ascending ethanol series, the enamel specimens were embedded in Araldite CY212 (AgarScientific, Stansted, UK). After decalcification of the remaining enamel with 0.1 M HCl, the samples were re-embedded in Araldite. The specimens were sectioned with an ultramicrotome (Ultracut E, Reichert, Bensheim, Germany), the ultrathin sections of the pellicle specimens were placed on pioloform-coated copper grids (Plano, Wetzlar, Germany) and contrasted with uranyl acetate and lead citrate. A TEM TECNAI 12 Biotwin (FEI, Eindhoven, The Netherlands) was used for transmission electron microscopy analysis at up to 49,000× magnification.

### Statistics

To determine statistical significant differences between groups, a student’s t-test was used with GraphPad.

## Results

### Calcium- and phosphate release

**Figure 2:**
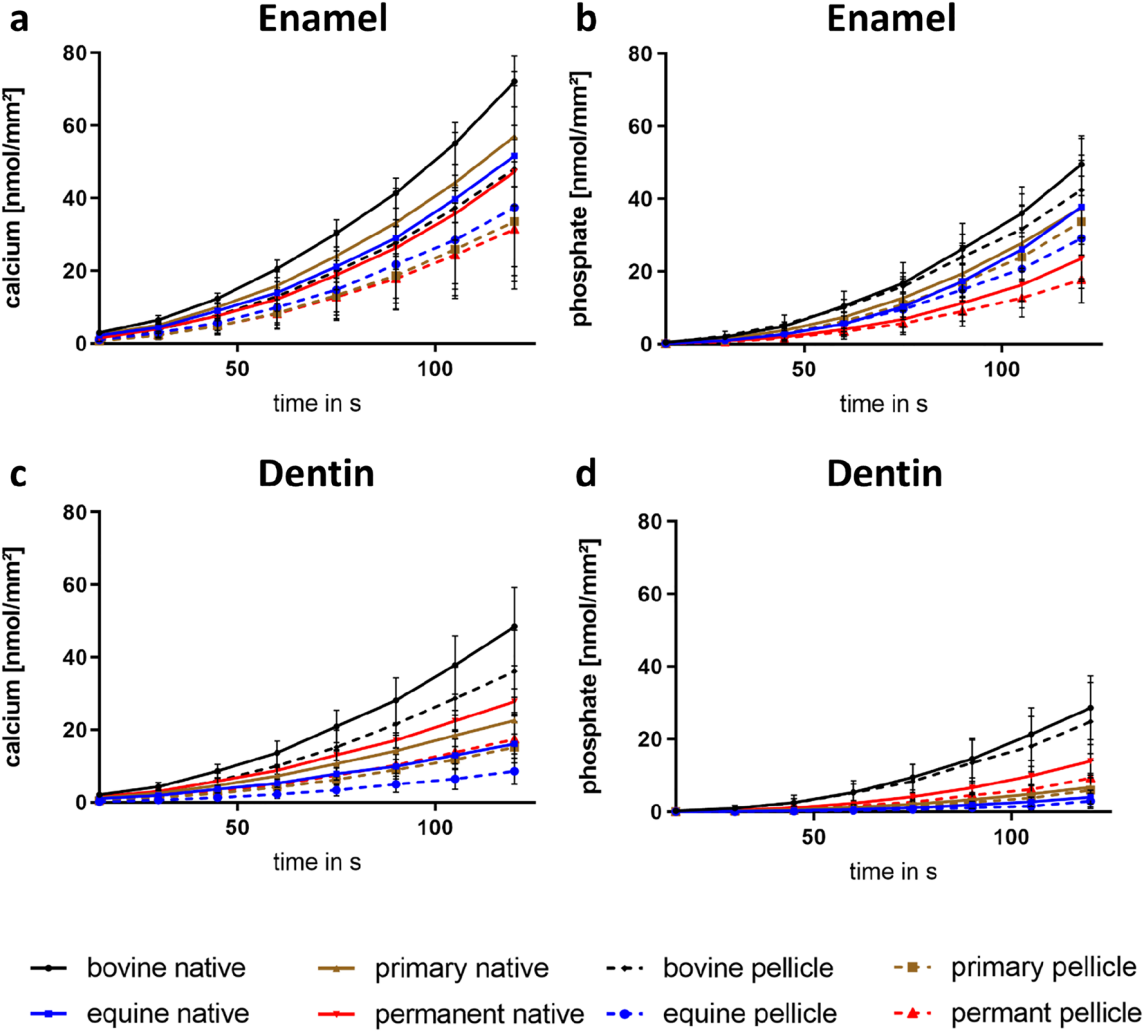
Kinetics of calcium (**a**,**c**) and phosphate (**b**,**d**) release from enamel (**a**,**b**) and dentin (**c**,**d**) samples of all species during 120 s incubation of native and pellicle-covered samples in HCl at pH 2.3. For all species, there was an increase in ion dissolution over the incubation time. A demineralisation-preventing effect of the pellicle was observed for all species. n = 4 subjects during pellicle formation with 3 hard tissue samples each, n = 8 native samples per hard tissue substance, mean values ± standard deviation.

**Figure 3:**
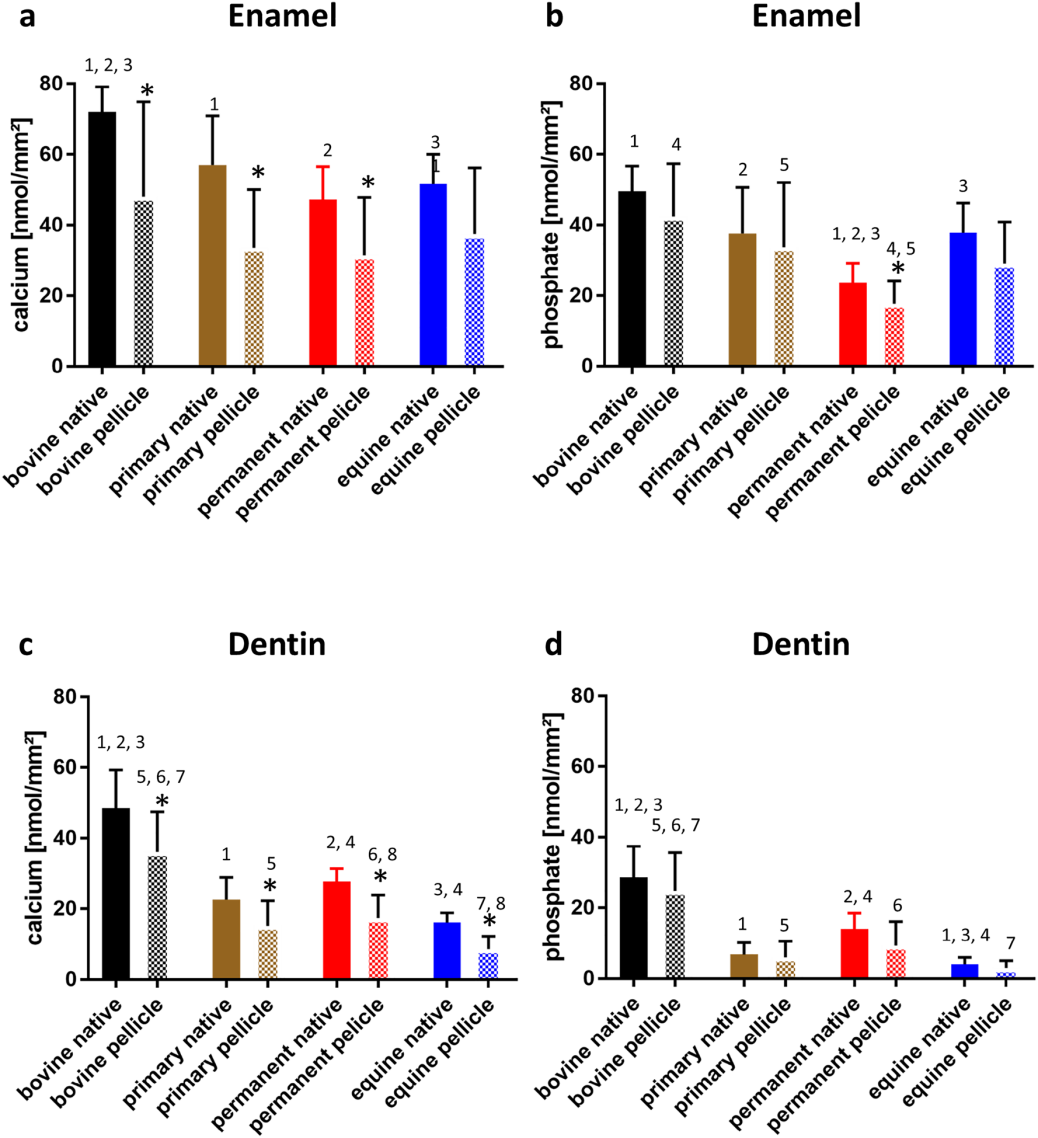
Cumulative calcium (**a**,**c**) and phosphate (**b**,**d**) release from enamel (**a**,**b**) and dentin (**c**,**d**) samples of bovine, human primary and permanent teeth and equine teeth after 120 s acid exposure with HCL at pH = 2.3 with and without 30 min pellicle formation (n = 4 subjects, 2 samples per hard substance and species). In situ pellicle formation reduced mineral release from enamel samples and, to a slightly lesser extent, from dentin samples of all species. Asterisks indicate significant differences between native and pellicle-formed samples (p < 0.05). Columns with the same number above are significantly different (t-test, p < 0.05).

In the enamel and dentin samples of all species, there was an increase in calcium and phosphate release with increasing exposure time at a pH of 2.3 (Fig. 2). In general, acid-induced calcium and phosphate release was higher in enamel samples than in dentin samples. Overall, a high standard deviation between replicates is observed. The formation of the 30-min-pellicles showed a significant protective effect against the erosive influence in all species, which was even more pronounced in the enamel samples than in the dentin samples (Figs. 2 and 3). However, the protective property of the pellicles was more pronounced for calcium release (significant for all species for enamel and dentin, except for enamel from equine teeth) than for phosphate release (only significant for enamel from human permanent teeth).

### Enamel samples

For native enamel samples, bovine samples showed significantly higher calcium release after 2 min of acid exposure than enamel samples from human or equine teeth (p < 0.0002). Phosphate release from native enamel samples was significantly higher in bovine samples than in human permanent teeth (p < 0.0001) and was significantly different between primary teeth and permanent teeth (p = 0.0208) and between human permanent teeth and equine teeth (p = 0.0189).

In pellicle-covered enamel samples, there were no statistically significant differences in calcium release between bovine, human and equine tooth samples. The phosphate loss of the pellicle-covered bovine enamel samples was significantly higher than that of the human permanent enamel samples. When comparing the human samples, the enamel of the primary teeth showed significantly higher phosphate losses than the enamel of the permanent teeth (p = 0.0305).

### Dentin samples

Native dentin samples showed significantly higher acid-induced calcium and phosphate release in the bovine samples compared to the human and equine dentin samples (p < 0.000). Calcium and phosphate release was also significantly higher in the human samples compared to the equine dentin (p = 0.0047). In the dentin samples, the protective effect of the pellicles was lower, as bovine dentin had significantly higher calcium and phosphate losses than human and equine dentin samples (p < 0.0001).

### SEM

**Figure 4:**
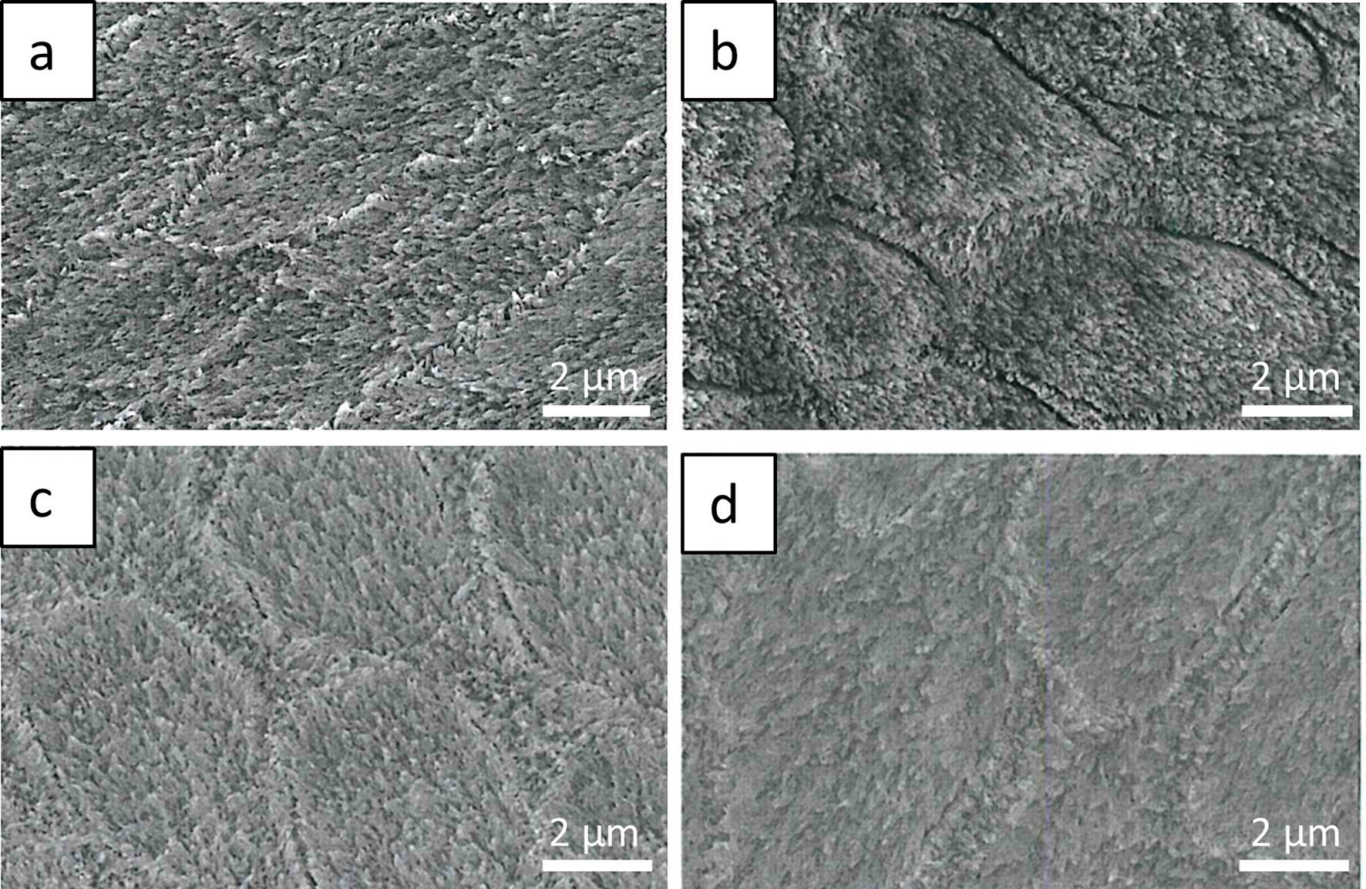
Scanning electron micrographs of acid-etched native enamel surfaces after 120 s incubation to HCl at pH = 2.3, (**a**) bovine enamel, (**b**) equine enamel, (**c**) primary enamel, (**d**) enamel of permanent teeth. The enamel crystallites are exposed in all species by the acid action. No structural differences were observed between the etched enamel samples of all species. Original magnification: 10,000-fold.

The scanning electron microscopic examination of the native enamel and dentin samples after acid exposure shows clear alterations of the surfaces. Both the enamel prism structure and the interprismatic substance are clearly visible in all species. The enamel crystallites are exposed following incubation in acid. No structural differences were observed between etched enamel samples of all species at the selected magnification (Fig. 4).

**Figure 5:**
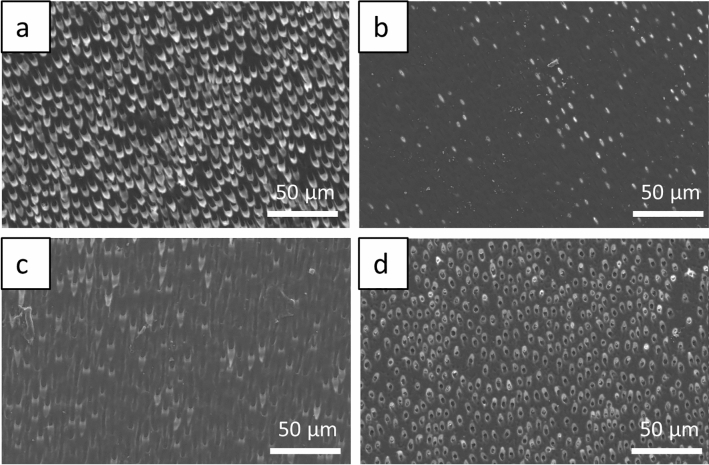
Scanning electron micrographs of acid-etched native dentin surfaces after 120 s incubation in HCl at pH = 2.3. (**a**) bovine dentin, (**b**) equine dentin, (**c**) primary dentin, (**d**) permanent tooth dentin. Comparing the dentin surfaces, there are structural similarities between bovine dentin and human permanent tooth dentin (diameter of dentin tubules ~ 2.5 µm). Equine dentin (**b**) is characterised by low density and irregular arrangement of dentin tubules (diameter < 2.0 µm). The dentin tubules of primary teeth are slightly larger (~ 3.0 µm) than those of permanent teeth (~ 2.5 µm). Original magnification: 500-fold.

When looking at the dentin surfaces, it is noticeable that there are great structural similarities between bovine dentin and the dentin of human permanent teeth. The diameter of the dentinal tubules of bovine samples and human permanent tooth samples is with ~ 2.5 µm approximately the same. Equine dentin (Fig. 5b) appears to be characterised by low density and irregular arrangement of dentin tubules compared to bovine and human permanent dentin (Fig. 5a/d). The diameter of equine dentinal tubules is < 2.0 µm. The dentinal tubules of primary teeth are slightly larger (~ 3.0 µm) than those of permanent teeth (~ 2.5 µm), but less densely arranged (Fig. 5c).

### TEM

**Figure 6:**
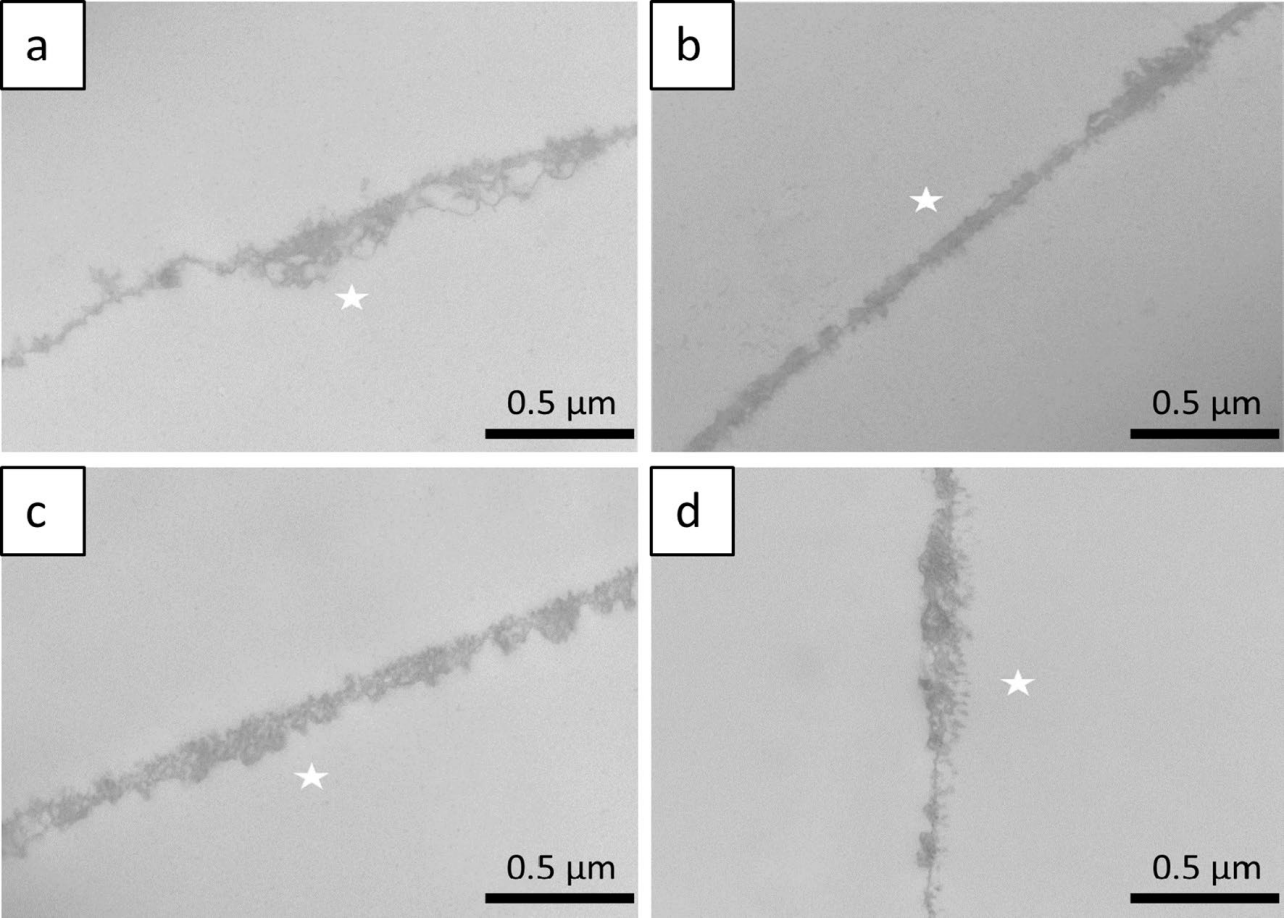
Representative TEM images of 2 h in situ pellicle samples on (**a**) bovine, (**b**) equine and (**c**) human primary and (**d**) permanent enamel. A comparable universal pellicle with a characteristic coherent protein structure is visible on all enamel surfaces. The former enamel site is marked with an asterisk as it was removed during the preparation process. Original magnification: 49,000-fold.

To investigate the ultrastructural characteristics of the pellicle formed on the enamel samples of human, bovine and equine teeth, TEM analyses were performed on 2 h in situ pellicle samples. All pellicle samples showed the characteristic thin but electron dense and continuous protein accumulation on the former enamel surface, the typical ultrastructure was quite similar in enamel samples of different species (Fig. 6a–d).

**Figure 7:**
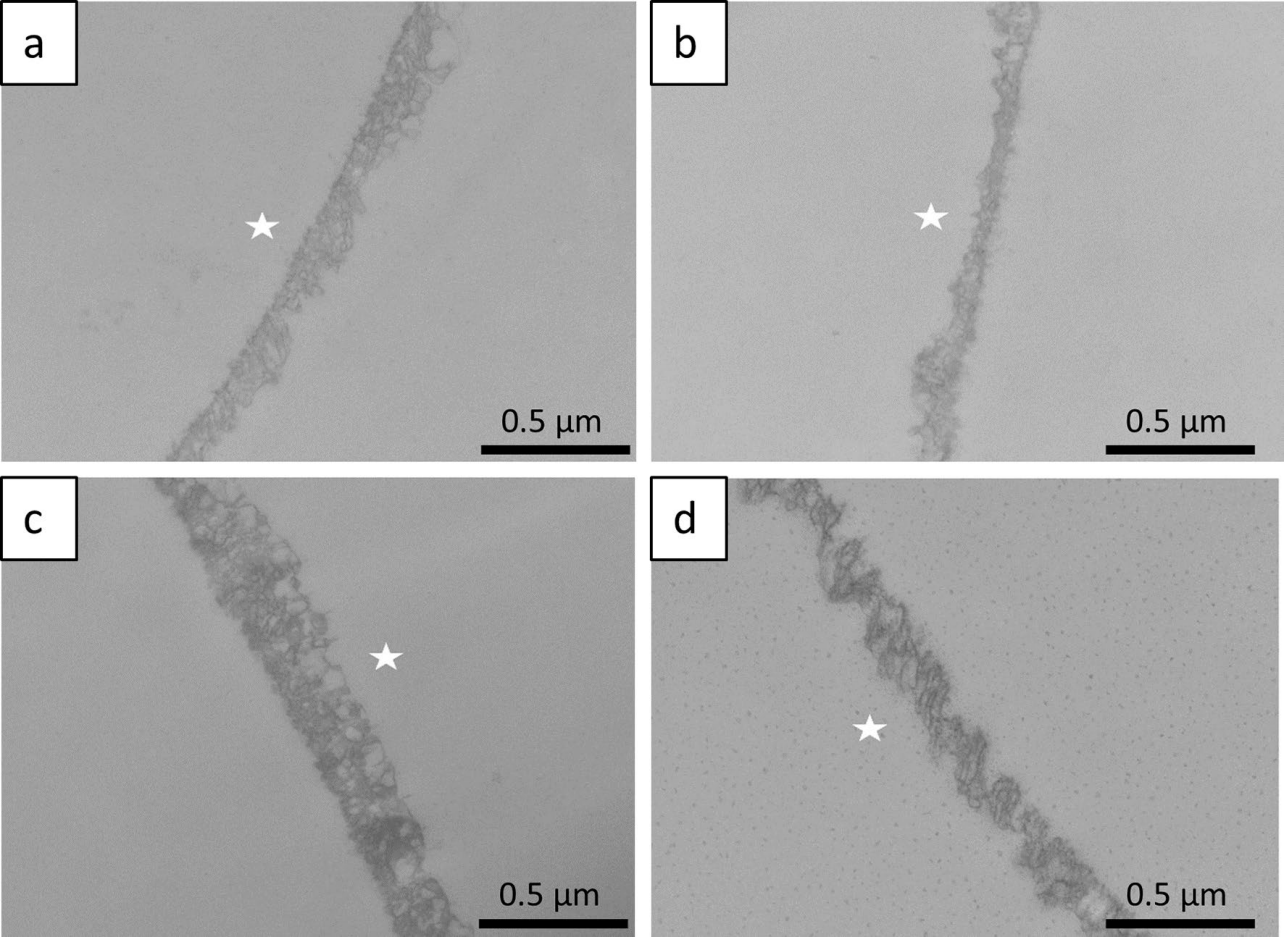
Incubation of the 30 min pellicle-coated enamel samples for 1 min in Coke Zero and subsequent oral exposure for a further 89 min resulted in partial protein desorption of the (**a**) bovine and (**d**) human permanent samples, whereas the equine samples showed only reduced thickness (**b**). The human primary samples showed acid-induced dissolution of the pellicle, resulting in a subsurface pellicle (**c**). The former enamel site is marked with an asterisk as it was removed during the preparation process. Original magnification: 49,000-fold.

After exposure of the 30 min in-situ pellicle to Cola Zero at pH 2.2 for 1 min and subsequent in-situ exposure for 89 min, all samples showed an altered pellicle structure, with the bovine and human permanent enamel samples showing evidence of partial protein desorption. In particular, the primary enamel samples showed dissolution of the pellicles, resulting in protein infiltration and a subsurface pellicle (Fig. 7a–d). The equine enamel samples showed the reduced pellicle thickness in comparison, but again no evidence of acid-induced pellicle dissolution could be detected.

## Discussion

In the present study, the demineralisation behaviour of the enamel and dentin of bovine, human and equine teeth with and without protective pellicles was compared for the first time using highly sensitive methods to test whether bovine and/or equine teeth are suitable alternatives to human dental hard tissues in the study of erosive processes. This provided reference data for future studies of demineralisation behaviour.

Many different methods are used in the literature to study erosive effects on enamel and dentin. In our study, as in many previous studies, photometric determination of calcium or phosphate release was chosen^2,3^. The advantage of these methods is that they can be used to study minimal erosive effects with short-term exposure to erosive substrates, even minimal erosive effects occurring within one minute can be quantified^16–18^. The detection limits are 7.3 µM for phosphate and 12.4 µM for calcium. Other methods for the detection of erosion-induced tooth structure defects, such as microhardness or surface profilometry, can only examine flat-parallel ground specimens, which precludes the examination of native hard substance specimens^19^. In addition, initial erosive tooth structure loss or dissolved tooth structure layers cannot be measured. Besides the photometric method, the quantification of calcium ions dissolved by erosion is also possible using highly sensitive atomic absorption spectroscopy^7^ or Raman spectroscopy^20^. However, these methods are very time consuming and costly as it requires the use of complex test solutions and/or expensive equipment. Furthermore, the photometric method used also allows the erosion kinetics to be recorded, as continuous measurements can be carried out due to the small volume of test solution removed from the incubation medium. Ultimately, this is a simple, highly sensitive and rapid test with uncomplicated sample preparation.

We decided to use a short in-situ pellicle formation and in-vitro acid exposure time to compare the demineralisation behaviour of tooth hard substances under clinically relevant conditions. The partial protective function of the enamel pellicle against erosive influences is already given after a pellicle formation time of 3 minutes^21,22^. This has previously been demonstrated with regard to calcium release and microhardness changes in tooth structure under erosive conditions^22^.

Our results showed that native bovine enamel samples were more susceptible to hydrochloric acid than the human and equine enamel samples. A possible explanation could be post-eruptive maturation of the enamel, as the bovine teeth were obtained from younger individuals than the human or equine permanent teeth. Posteruptive enamel maturation is a process that leads to changes in tooth hard tissues after the tooth erupts into the oral cavity. Over time, the properties of enamel change as a result of water loss, reduction in organic matrix components and changes in crystal structure^23^. Minerals are absorbed from saliva or food, so the mineral content of enamel changes over time. In humans, fluoride compounds from oral care products in particular are substituted, resulting in reduced acid solubility of enamel^23,24^. In our study, equine enamel has comparable acid solubility to human enamel. Although posteruptive enamel maturation in horses is not due to fluoride substitution, minerals such as calcium and phosphorus can be integrated from saliva into the enamel over time by chewing horse feeds such as hay, silage or mineral feeds and alter the acid solubility of equine enamel^25^.

Interestingly, the formation of a 30 min in situ pellicle compensated for this susceptibility to the extent that no significant differences in acid-induced calcium release were observed between bovine, human and equine enamel. The basic erosion protection of the pellicle is due to the fact that the interaction of the acid with the tooth surface is delayed^26,27^. The most important role in maintaining mineral homeostasis is played by calcium-binding pellicle precursor proteins such as acidic proline-rich proteins, statherin and histatins. They are thought to maintain high concentrations of calcium ions within the pellicle and closer to the apatite surface, thereby reducing erosive demineralisation processes^26–28^. The finding that acid-induced phosphate release from enamel is significantly higher in bovine enamel than in human enamel, despite pellicle formation, should not be overstated at this point.

In the dentin samples of all species, the erosion protective effect of the pellicle was less clearly demonstrated, in agreement with previous in vitro and ex vivo studies^6,7,29,30^. When comparing the pellicle-covered dentin samples, both calcium and phosphate release from bovine dentin are significantly higher than from human and equine dentin. The fact that significant differences in mineral release remain between human permanent dentin and equine dentin despite pellicle formation demonstrates the significantly reduced protection against erosive demineralisation of pellicle on dentin surfaces, which may be related to its higher porosity and solubility^6,26^. A recent study using transmission electron microscopy has also shown that 30-min pellicles are not effective in protecting dentin from erosion, which is in contrast to the data for enamel pellicles^30^.

When comparing primary and permanent human teeth, we found a higher release of phosphate in particular, but also calcium, in primary enamel compared to permanent enamel, both in native samples and after pellicle formation. However, the opposite trend was observed in dentin samples, where erosion was higher in permanent teeth compared to primary teeth. Although these observations were not always significant, this suggests a greater erosion of primary enamel compared to enamel from permanent human teeth, but conversely a greater erosion of dentin from permanent teeth compared to primary teeth. Similar observations have previously been made in a long-term in vitro erosion study (5–15 days) following exposure of primary and permanent enamel and dentin to low pH fruit drinks, but the results were not statistically significant^31^. Structural differences in enamel between primary and permanent teeth explain these results and confirm the increased susceptibility of primary teeth to erosion reported in the literature^32,33^. Primary enamel is less mineralised and therefore has a lower microhardness than permanent enamel^34,35^. Dental enamel also contains more water and has a higher permeability^36,37^. This has also been demonstrated by TEM. After incubation of the 30-min pellicles in Coke Zero and subsequent intraoral exposure of the samples, a clear dissolution of the pellicles with signs of demineralisation was observed in the primary enamel samples, so that a subsurface pellicle was detectable. This also demonstrates the lower degree of mineralisation of primary enamel with increased porosity and susceptibility to erosive enamel defects compared to human permanent teeth and bovine and equine samples. It would also be of interest to repeat the in situ experiments with children as participants since a previous study suggested different erosive protective effects by pellicles formed from saliva by adults or children at least under in vitro conditions^38^.

The observation that primary dentin is less susceptible to erosive demineralisation than permanent dentin was not expected, as structural differences in dentinal tubule size and inter-tubular dentin would suggest the opposite. Primary dentin has a greater number of larger diameter dentinal tubules than permanent dentin^39^. Calcium and phosphate concentrations in peritubular and intertubular dentin are lower in primary teeth than in permanent teeth, leading to a more rapid progression of acid-induced demineralisation^39^. On the other hand, the higher organic content of primary dentin may also limit the diffusion of ions into and out of the demineralised surface. This reduces the progression of dentin erosion in primary teeth^40^. A more detailed investigation of the erosion behaviour of primary dentin compared to permanent dentin would be important.

Electron microscopy is well established for the study of erosion processes on dental hard tissue^17^. The electron microscopic images illustrate basic structural features. When comparing the acid-exposed enamel samples, no structural differences between the species were found by SEM. For the dentin samples, it was confirmed that coronal bovine dentin and dentin from human permanent teeth are structurally similar, whereas equine dentin is characterised by a smaller tubule diameter and a less dense arrangement. This may be due to the constant wear and tear of equine teeth throughout their lives. However, SEM does not allow quantification of demineralisation processes, which would be of interest for future studies comparing the erosion susceptibility of the dental hard materials used. TEM examination of the 2 h pellicle ultrastructure visualises a characteristic pellicle on the enamel surfaces of human, bovine and equine teeth, confirming the uniformity of pellicle formation. This would also be in line with a study showing that the proteomic profile of the in situ formed pellicle on human or bovine enamel is very comparable^41^.

When considering whether bovine or equine teeth are a suitable alternative to human teeth for the study of erosive processes, several factors must be taken into account. Firstly, it is important that the general elemental composition is similar. A previous study has shown that the composition of bovine and human teeth is similar, particularly in terms of calcium and phosphate content^42^. Another study also suggested similar elemental levels of calcium and phosphate in the dentin of human and equine teeth^43^.

Another issue is the availability and consistency of tooth material. Bovine teeth are readily available as a by-product of abattoirs. Cattle are fed a uniform diet, are exposed to the same environmental conditions and the teeth obtained from the animals are of a similar age. Horse teeth, on the other hand, are less readily available because the animals are not slaughtered at a specific age by default. Therefore, the availability of equine teeth is less predictable and the age of the animals varies widely, as in our study where equine teeth from 15 to 30 year old horses were used. Last but not least, the anatomical peculiarities of equine teeth should also be taken into account, as there is a complex arrangement of enamel-dentine cement structures and post-eruptive maturation of the outer enamel^13^. It is not uncommon for incisor crowns to be curved and for the enamel to be covered with a layer of cementum, unlike human and bovine enamel^44^. This makes it difficult to obtain homogeneous enamel samples. According to our data, the erosive properties of equine teeth are closer to those of human teeth than those of bovine teeth, although the relative differences are comparable. The data therefore suggest that both bovine and equine teeth are good substitutes for human teeth, especially since the differences in calcium and phosphate release in the enamel of human and bovine teeth are no longer statistically significant after the formation of the 30-min pellicle. As mentioned above, a major advantage of bovine teeth is their relative uniformity with respect to age and diet. It is important to note, however, that we had a small sample size, which is common in basic dental research and yet provides important insights into biological conditions.

However, the availability of human teeth is limited as they are mostly obtained from routine tooth extractions as part of dental treatment. As a result, donor age, diet and fluoride intake are highly variable and tooth extraction is often due to caries. In addition, only small samples can be obtained from human teeth.

In conclusion, bovine enamel appears to be somewhat more susceptible to erosive processes, which should be taken into account in future studies. Nevertheless, due to its availability and uniformity compared to equine teeth, it is a good alternative to human tooth specimens for in situ studies.

## Conclusions

- Erosion of bovine enamel or dentin is greater than that of human or equine hard tooth tissue.
- Erosion of human primary tooth enamel is greater than that of permanent teeth, whereas dentin shows the opposite behaviour.
- Eroded equine dentin appears more irregular than human or bovine materials.
- Both bovine and equine enamel or dentin are suitable substitutes for hard dental tissue from human teeth.
- The data from this study can serve as an important reference for future studies on the erosion and demineralisation of hard dental tissues.

## Data Availability

All data generated or analysed during this study are included in this published article (and its Supplementary Information files).
